# Overdose Alert and Response Technologies: State-of-the-art Review

**DOI:** 10.2196/40389

**Published:** 2023-02-15

**Authors:** Alberto Oteo, Hadi Daneshvar, Alexander Baldacchino, Catriona Matheson

**Affiliations:** 1 DigitAS, Populations and Behavioural Science Division School of Medicine University of St Andrews St Andrews, Fife United Kingdom; 2 Salvation Army Centre for Addiction Services Faculty of Social Sciences University of Stirling Stirling United Kingdom; 3 NHS Fife Addiction Services Leven United Kingdom; 4 Faculty of Social Sciences University of Stirling Stirling United Kingdom

**Keywords:** drug overdose, technology, opioids, telemedicine, mobile health, mHealth, apnea, sensor, naloxone, mobile phone

## Abstract

**Background:**

Drug overdose deaths, particularly from opioids, are a major global burden, with 128,000 deaths estimated in 2019. Opioid overdoses can be reversed through the timely administration of naloxone but only if responders are able to administer it. There is an emerging body of research and development in technologies that can detect the early signs of an overdose and facilitate timely responses.

**Objective:**

Our aim was to identify and classify overdose-specific digital technologies being developed, implemented, and evaluated.

**Methods:**

We conducted a “state-of-the-art review.” A systematic search was conducted in MEDLINE, Embase, Web of Science, Scopus, ACM, IEEE Xplore, and SciELO. We also searched references from articles and scanned the gray literature. The search included terms related to telehealth and digital technologies, drugs, and overdose and papers published since 2010. We classified our findings by type of technology and its function, year of publication, country of study, study design, and theme. We performed a thematic analysis to classify the papers according to the main subject.

**Results:**

Included in the selection were 17 original research papers, 2 proof-of-concept studies, 4 reviews, 3 US government grant registries, and 6 commercial devices that had not been named in peer-reviewed literature. All articles were published between 2017 and 2022, with a marked increase since 2019. All were based in or referred to the United States or Canada and concerned opioid overdose. In total, 39% (9/23) of the papers either evaluated or described devices designed to monitor vital signs and prompt an alert once a certain threshold indicating a potential overdose has been reached. A total of 43% (10/23) of the papers focused on technologies to alert potential responders to overdoses and facilitate response. In total, 48% (11/23) of the papers and 67% (4/6) of the commercial devices described combined alert and response devices. Sensors monitor a range of vital signs, such as oxygen saturation level, respiratory rate, or movement. Response devices are mostly smartphone apps enabling responders to arrive earlier to an overdose site. Closed-loop devices that can detect an overdose through a sensor and automatically administer naloxone without any external intervention are still in the experimental or proof-of-concept phase. The studies were grouped into 4 themes: acceptability (7/23, 30%), efficacy or effectiveness (5/23, 22%), device use and decision-making (3/23, 13%), and description of devices (6/23, 26%).

**Conclusions:**

There has been increasing interest in the research and application of these technologies in recent years. Literature suggests willingness to use these devices by people who use drugs and affected communities. More real-life studies are needed to test the effectiveness of these technologies to adapt them to the different settings and populations that might benefit from them.

## Introduction

### Background

There were an estimated 128,000 deaths related to drug use disorders worldwide in 2019, with illicit and licit opioids accounting for over two-thirds of these deaths [1]. Countries in North America and Northern Europe have particularly high rates of fatal drug overdose (OD), with OD deaths in the United States surpassing 100,000 for the first time in 2021 [2] and Canada seeing a 98% increase in opioid ODs during the first year of the COVID-19 pandemic (April 2020 to March 2021) to 7224 deaths [3]. Drug-related deaths (DRDs), a broader term used to compare rates of death because of drug misuse in Europe, which mostly implies drug OD, have also been increasing alarmingly in some European countries such as the United Kingdom, particularly in Scotland, which has a DRD rate of 327 per million aged 15 to 64 years in 2020 [4], more than 20 times the average rate in Europe [5]. Most of these deaths (89%) are attributed to opioids, often in combination with other sedatives. The appearance of potent synthetic opioids in illicit drug markets during the last decade has worsened the situation, especially in North America [2,6-8].

It is common for people who use drugs, especially those at risk of OD such as people who use opioids, to use their drugs alone [9,10], which in turn increases the risk of fatal OD. This factor is key as opioid ODs in particular can be reversed through the timely administration of naloxone, a highly effective opioid antagonist [11]. The time window between the first symptoms of OD, such as reduced respiratory rate and low blood oxygen (saturation of peripheral oxygen [SpO_2_]) level, and death ranges from a few minutes to several hours, although controlled studies with prescribed diamorphine have found that most severe adverse events, such as acute respiratory depression, appear within a few minutes of administration [12,13]. If medical responders arrive within that window, the person is more likely to survive.

Several countries have now implemented naloxone distribution programs where not only medical personnel can assist opioid OD with naloxone, but also service users and their families are able to use it when necessary [11,14,15]. In these programs, training and naloxone kits are provided to people who use opioids, family members, service providers, and other people who may be able to witness and quickly assist an OD. These programs have proven to save many lives [11]. A systematic review and meta-analysis of international studies conducted by Burton et al [16] found levels of ownership of take-home naloxone of 57% on average in at-risk people who inject drugs but a much lower level of carriage (20%).

Social isolation and marginalization increase solitary drug use, and solitary use increases the risk of OD death [17]. Quinn et al [18] further showed that nearly three-quarters of OD deaths in people who use opioids had no evidence of naloxone administration. Therefore, if people who use opioids are alone when they use drugs, it is unlikely for somebody carrying naloxone to be able to help them. To overcome this challenge, different creative ideas are being put into place or tested. For example, people who use drugs and other community members have been applying an intervention called “spotting” [19], where someone’s drug consumption session is monitored by someone else via telephone or video call. This community-driven intervention is able to reduce the risk of overdosing alone but relies heavily on human intervention. Other researchers are attempting to develop technologies to overcome the burden of intensive human support. The development of technologies that can detect OD and alert first responders in a timely manner even when the person is using drugs alone can be an effective way to reduce the number of DRDs, but research on this topic is still scarce.

During the last decade, and especially during the COVID-19 pandemic, telehealth interventions have been implemented to improve the coverage of addiction services and reduce harms [13-15]. Some of the technologies involved in these interventions are specifically aimed at detecting drug OD and alerting responders as well as facilitating the timeliness of the response. The development of OD detection and alert technologies (ODART) is a very recent field of research and practice with no clear overview of the technologies that are being used or developed to prevent drug OD, the conditions in which they have been applied, or their characteristics.

### Objectives

This study aimed to identify OD-specific digital technologies being developed, implemented, and evaluated to classify them based on their characteristics and functionality and to describe studies that have been conducted and classify them thematically. Where possible, we assessed the advantages and limitations of these technologies in the real world.

## Methods

### Study Design

We conducted a “state-of-the-art review, ” which, according to Grant and Booth [20], is a search of recent literature that addresses more contemporary matters when compared with other combined retrospective and current approaches. This approach examines current knowledge, offers new perspectives, and highlights areas for further research.

The nature of this review is mainly descriptive. We anticipated that the studies would not be sufficiently homogeneous to conduct a quantitative synthesis. The included studies were classified by type of technology and its function, year of publication, country of study, target population, study design, findings, and limitations.

The definition of the target condition, drug OD, was established as “the excessive dosing of a substance administered so that it causes medical complications, including death.” However, given the different definitions that the term might have, we considered studies that used different definitions as long as they were in line with ours. The focus of research in this field is oriented toward opioid OD given that it is the major cause of OD and that resuscitation with naloxone makes ODART especially suitable for opioids. However, we included any drug OD as we were interested in any technologies that can be used to improve OD detection and response. Even though it is implied that most ODs are caused by opioid use, technologies that are able to detect and alert of OD caused by any other drugs would be included in the scope of this study.

We excluded devices that measured vital signs and could be used as ODART but were not specifically being developed or marketed as such as this would result in a large and nonspecific list. For the same reason, we also excluded devices that were not clearly in use or development or whose development had been discontinued. Similarly, we excluded devices that had only been documented in patents.

### Search Strategy

A systematic search was carried out on June 3, 2021. Searches were conducted in MEDLINE, Embase, Web of Science, Scopus, ACM Digital Library, IEEE Xplore Digital Library, and SciELO (Multimedia Appendix 1). The search included terms related to telehealth and digital technologies, drugs, and OD and papers published since 2010. We also searched references from articles and scanned the gray literature, defined as information that is produced outside of traditional academic publishing and distribution channels and is not peer-reviewed, including industry websites and forums and government grant registries. We included nonacademic search engines for products that have been or are being developed or that are being used but have not been the subject of academic publication. We contacted key actors, including the companies and researchers developing the technologies, to obtain more information. The complete search strategy can be found in Multimedia Appendix 1 and was published in the PROSPERO web-based database for systematic reviews [21]. The review was conducted and documented in line with the PRISMA-P (Preferred Reporting Items for Systematic Reviews and Meta-Analyses extension for Protocols) [22] checklist. A repeat search was conducted close to submission on April 10, 2022, to identify very recent publications. We followed the same procedure with the exclusion of the search term “poison*,” which in the first run resulted in a large number of papers relating to topics other than those in the scope of our review and did not result in any included paper that the term “overdose” would have missed.

We included all studies that described technologies for humans who use drugs and are at risk of OD. We included all methodologies (ie, not restricted to randomized controlled trials) and any language of publication. We excluded studies published before January 2010 and studies dealing with technologies not specifically addressing OD (eg, that only provided advice or treatment or measured other aspects of addiction such as withdrawal symptoms). We excluded mentions in commercial websites and government grants of devices that already appeared in academic papers to avoid duplication.

One reviewer (AO) conducted searches, collated results, and deduplicated them using EndNote X9 (Clarivate Analytics) and Rayyan (Rayyan Systems Inc), a specialized software for systematic reviews [23]. In total, 2 researchers (AO and HD) reviewed titles and abstracts against the eligibility criteria in parallel. Discrepancies were reviewed by the rest of the team, and those papers were classified based on consensus. A total of 2 reviewers (AB and CM) provided quality assurance by independently checking a random selection of 20 titles and abstracts: 10 selected papers and 10 papers rejected by AO and HD. Areas of disagreement were discussed by the team. The final data extraction table was reviewed separately by all the authors, and there was agreement on the inclusion of the final papers. Reasons for exclusion were mostly because the papers did not fall within the scope of the review, either because they did not refer to drug ODs but to accidental poisonings that were not drug-related or because, if referring to use of opioids or OD prevention, they did not refer to ODART as technologies to detect and alert responders in case of OD.

### Quality Appraisal and Study Details

Given that we expected a low number of studies and anticipated that many of them would be qualitative, proof-of-concept, or efficacy studies, we put no restrictions on the type of study. This is a common feature of state-of-the-art reviews where, instead of using quality assessment as an inclusion criterion, studies are considered for inclusion based on their relevance [20]. Therefore, instead of using quality assessment tools, we assessed the sample sizes, data collection methods, and perceived limitations of each study, although we did not use any formal quality assessment tool.

### Data Analysis

Paper characteristics, including year of publication, country of study, target population, function of technology, study design, and findings, were entered into a spreadsheet. A thematic analysis led by AO and HD and reviewed by the other authors was used to group studies, compare them, and analyze their findings and conclusions.

We classified the studies according to three parameters: (1) type of technologies described in the studies, (2) focus of studies in terms of what aspect of the technology at hand was under scrutiny, and (3) findings of the studies.

## Results

### Overview

**Figure 1:**
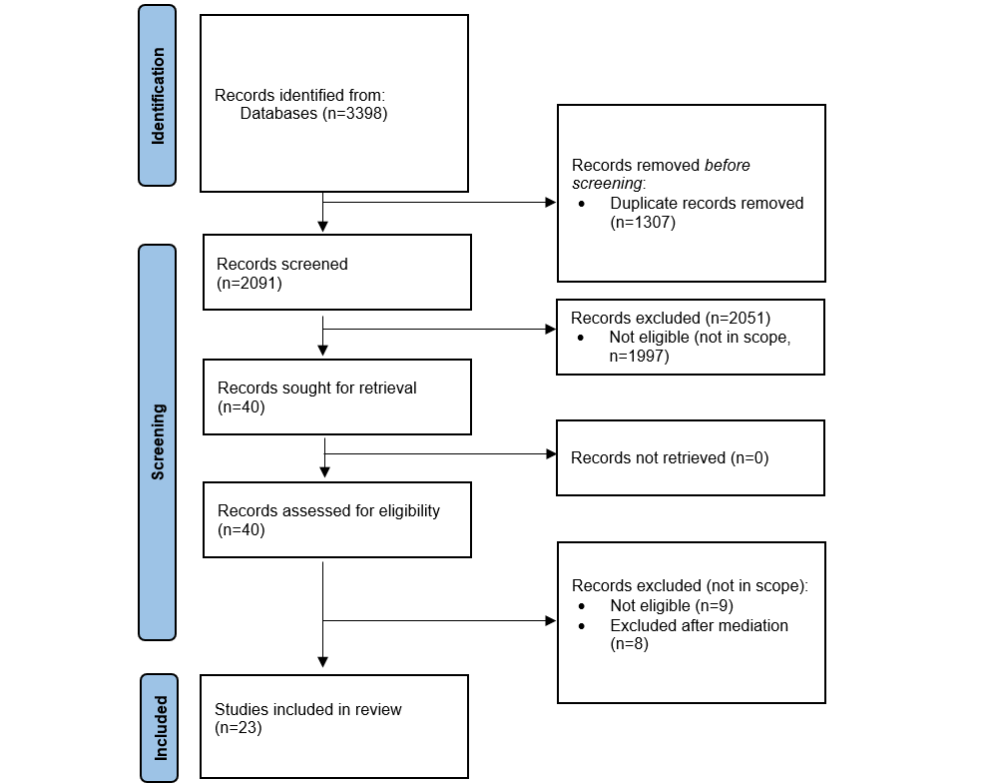
PRISMA-ScR (Preferred Reporting Items for Systematic reviews and Meta-Analyses extension for Scoping Reviews) flow diagram.

As shown in Figure 1, from an initial 2091 identified studies, 40 (1.91%) were sought for full-text retrieval, and 23 (58%) of them were finally included in our review.

The included papers were published between 2017 and 2022, with a marked increase since 2019 (20/23, 87% of the papers were published since 2019). This suggests that there is a growing body of research on ODART, probably driven by increases in drug ODs in North America and parts of Europe and by technological improvements as well as accessibility to these technologies.

### Overview of Papers: Study Designs

Of the 23 selected papers, 17 (74%) studies conducted primary research [24-40] using mixed methods [26,29,38], qualitative [27,28,41], and quantitative research [24,25,30-37,40]; 1 (4%) was a protocol for a clinical trial study [42]; 2 (9%) described proof-of-concept devices [41,43]; and 4 (17%) were literature reviews [44-47]. All studies were conducted in North America (United States and Canada) except for one that was based in Israel but used data from the United States [37]. In the 57% (13/23) of primary research studies, sample sizes ranged from 11 [31] (N=3 considering tests done with mice [25]) to 1061 [36]. The 9% (2/23) of studies that used data from emergency medical services (EMS) registries had sample sizes of 19,437 [37] and 200 [30].

In addition to peer-reviewed papers, we found 3 grants awarded to groups researching closed-loop ODART devices [39,48,49]. Through our gray literature search, we also found some devices that are already operating or were being developed specifically as ODART [50-56].

### Types of Technologies

#### Overview

We classified technologies into 3 main groups according to their main function, namely, “OD alert,” “OD response,” and “combined OD alert and response.” These are shown in Tables 1-3. Multimedia Appendix 2 [24-38,40-47] displays more detailed information about each study (ie, intervention, function, focus, device, theme, and study design). Although there is frequently a degree of overlap between the first 2 groups, as all devices ultimately need to combine OD alert and response, this classification is based on the main focus of the study. Some papers are repeated in different groups as they may describe several devices, so the total number of papers listed (30) is greater than the number of selected papers (23).

**Table 1:** Studies on overdose (OD) detection devices (N=9).

Author, year	Intervention	Focus	Device	Theme
Tsang et al [26], 2021	Different smartphone-based solutions to prevent OD	Willingness to use mobile phones for monitoring applications to mitigate OD; also asked about the app to report adulterated drug supply and receive OD alerts from others	Hypothetical app using smartphone cameras to monitor breathing while using drugs	Acceptability
Ahamad et al [36], 2019	Wearable biosensors	Willingness of people who use drugs to wear a device (skin patch) that can detect and alert others of an OD	Hypothetical skin patch sensor for OD detection	Acceptability
Carlile and Sunshine [33], 2019	Smartphone sensor and alert	Testing opioid users’ ability to turn off a smartphone simulation of an OD alarm and survey on acceptance of false alarms	Prototype smartphone OD alert app that would send a prompt to check on user alert	Acceptability; ability to turn off false alarms
Nandakumar et al [35], 2019	Smartphone sonar sensor	To present algorithms that run on smartphones and unobtrusively detect opioid OD events and their precursors	Sonar-based smartphone sensor (Second Chance)	Efficacy of tool (experiment)
Singh et al [31], 2019	Wearable sensor	To leverage accelerometer and blood volume pulse measurements from a wearable biosensor and use machine learning for the novel problem of collaborative nonadherence detection in opioid surveillance	Wrist-mounted sensor (Empatica E4)	Effectiveness of tool; diagnostic accuracy in detecting cheating
Roth et al [32], 2021	Wearable sensor	Looking at usability of wearable patch attached to clothing measuring respiratory rate and motion; hours of recording per day and comparison of recording based on types of users	OD detection skin patch sensor (Spire)	Feasibility; potential for sensor to be worn by people who use drugs and to record appropriately
Beaulieu et al [44], 2020	AI^a^ interventions for opioid use	Review of the gray literature to identify AI interventions specific to opioid use disorders being developed, implemented, and evaluated	Sonar-based smartphone sensor (Second Chance); wrist-mounted heartbeat, motion, skin electrical conduct, and temperature sensor (Empatica) but as withdrawal, not OD, detector	Description of technologies
Fairbairn et al [45], 2017	Different interventions, including apps and sensors	Overview of trends in opioid use in North America and proposing potential solutions	Mentions that a pilot study to investigate the feasibility of a novel mobile device to monitor vital signs in opioid-injecting individuals is currently underway in Vancouver, British Columbia, Canada (the Mobile Monitoring of Vital Signs in Opioid Users [MOVE] 2015).	Description of technologies
Goldfine et al [46], 2020	Digital interventions for substance use; wearable sensors and wireless technology	To evaluate the advances in wearable and other wireless mHealth^b^ technologies in the treatment of substance use disorders	Mentions sonar-based smartphone sensor (Second Chance, Nandakumar et al [35]) and wrist-mounted sensor (Empatica E4) and cites Ahamad et al [36] on hypothetical sensor	Description of technologies

^a^AI: artificial intelligence.

^b^mHealth: mobile health.

**Table 2:** Studies on overdose (OD) response devices (N=10).

Author, year	Intervention	Focus	Device	Theme
Marcu et al [34], 2020	Smartphone app to connect those who witness an OD with volunteer responders	Piloting a smart app to connect potential responders with each other; UnityPhilly is intended to create a network of people who use drugs as well as other members of the local community who report that they have not had any nonmedical opioid use	Smartphone OD response app (UnityPhilly)	Feasibility
Tukel et al [30], 2020	Drones for naloxone delivery	Comparing time required for a drone carrying naloxone to traverse various distances with the time required for ambulances to traverse similar distances while responding to the scene of actual or suspected opioid ODs	Modified DJI^a^ “Inspire 2” drone to carry naloxone	Effectiveness of tool
Khalemsky and Schwartz [37], 2017	Responder app community response based on geolocation app	Simulation of different emergency responses, among which was naloxone provision for OD; comparing a simulation of time to respond based on real-world data of community vs EMS^b^ response based on different parameters and their probability	Hypothetical smartphone OD response	Effectiveness; simulation of emergency response community effectiveness compared with EMS
Ataiants et al [29], 2021	Smartphone app to connect those who witness an OD with volunteer responders	Identification of heuristics that determine whether someone with a response app will signal an OD or be alerted of OD episodes based on need for assistance and contextual information	Smartphone OD response app (UnityPhilly)	Use or decision-making
Marcu et al [27], 2019	Acceptability of smartphone apps for facilitating layperson naloxone administration during opioid ODs	User requirements for a smartphone app to coordinate layperson administration of naloxone during an opioid OD	Hypothetical smartphone OD response and exchange of information on dangerous drug supply	Acceptability
Vilardaga et al [47], 2020	Opioid-related smartphone apps (no intervention)	Characterize the purpose, audience, quality, and popularity of opioid-related smartphone apps	Smartphone OD response app (NaloxoFind and UnityPhilly)	Description of technologies; quality evaluation
Schwartz et al [40], 2020	Smartphone apps to connect those who witness an OD with volunteer responders	To investigate whether equipping community members, including people who use drugs, with a smartphone app enabling them to signal and respond to suspected ODs would support naloxone administration in advance of EMS	Smartphone OD response app (UnityPhilly)	Effectiveness of tool (real life)
Tsang et al [26], 2020	Different smartphone-based solutions to prevent OD	Willingness to use mobile phones for monitoring applications to mitigate OD; also asked about app to report tainted drug supply and receive OD alerts from others	Hypothetical app to alert bystanders with naloxone	Acceptability
Fairbairn et al [45], 2017	Different interventions, including apps and sensors	Overview of trends in opioid use in North America and proposing potential solutions	Smartphone OD response app (Beacon Dispatch by Trek Medics)	Description of technologies
Goldfine et al [46], 2020	Digital interventions for substance use; wearable sensors and wireless technology	To evaluate the advances in wearable and other wireless mHealth^c^ technologies in the treatment of substance use disorders	Mention of the paper by Dhowan et al [25] on A2D2 closed-loop device	Description of technologies

^a^DJI: Dà-Jiāng Innovations.

^b^EMS: emergency medical services.

^c^mHealth: mobile health.

**Table 3:** Studies on combined alert and responder devices (N=11).

Author, year	Intervention	Focus	Device	Theme
Bristowe et al [42], 2021	Supervised consumption service	Protocol for piloting a telephone-based supervised opioid consumption service	Supervised consumption line	Acceptability
Imtiaz et al [43], 2021	Closed-loop device formed by a sensor and nalmefene injector	Presents design of the closed-loop device with sensor for SpO_2_^a^ and subsequent release of nalmefene when SpO_2_ goes <90%	Wearable noninvasive closed-loop device	Description of technologies
Chan et al [24], 2021	Closed-loop device formed by a sensor and naloxone injector	Presented proof of concept and evaluated in 2 environments: SIF^b^ and hospital environment simulating opioid-induced apnea in healthy participants	Closed-loop wearable injector system that measures respiration using a pair of on-body accelerometers; administers naloxone subcutaneously upon detection of an apnea	Evaluation of accuracy and efficacy of device
Kanter et al [38], 2021	Wearable sensors that detect OD^c^ and prompt an automated response; different functionalities offered but with special interest in closed-loop device that deploys naloxone	Willingness of people who use drugs to wear a device (wristband) that can detect an OD and prompt a response; different options are given and open questions allow for respondents’ input	Hypothetical wearable to detect an OD and elicit response with different options	Acceptability
Bardwell et al [28], 2021	Alarm button to alert staff in building	Uses for OD alert device by women in supported housing	OD response button (Brave Button)	Use or decision-making
Bivens [41], 2018	Smartphone app to use naloxone	To extend PXD^d^ to include community-based and technology-based contexts of use by analyzing 2 case examples; next, to discuss implications of community-based and technology-based PXD within communities of people who use opioids, critiquing each method and suggesting 4 contexts of use-heuristic categories to consider when designing health communication information for users in these contexts	OD detection skin patch sensor (Spire) and OD response smartphone app (OD Help)	Description of technologies; implications of community-based and technology-based contexts
Dhowan et al [25], 2019	Closed-loop device formed by a sensor and naloxone injector	Presented proof of concept and tested leakage in laboratory simulation (leakage with saline solution) and release capacity in mice	Subcutaneously placed device can be activated using an externally applied time-varying magnetic field from a wearable device; the device would be paired with an ECG^e^ and respiratory rate sensor	Proof of concept; testing of release capacity in mice
Tsang et al [26], 2021	Different smartphone-based solutions to prevent OD	Willingness to use mobile phones for monitoring applications to mitigate OD; also asked about app to report tainted drug supply and receive OD alerts from others	Smartphone OD alert and response app	Acceptability
Fairbairn et al [45], 2017	Different interventions, including apps and sensors	Overview of trends in opioid use in North America and proposing potential solutions	Smartphone supervised consumption app (OD Help)	Description of technologies
Goldfine et al [46], 2020	Digital interventions for substance use; wearable sensors and wireless technology	To evaluate the advances in wearable and other wireless mHealth^f^ technologies in the treatment of substance use disorders	Mention of the A2D2 closed-loop device by Dhowan et al [25]	Description of technologies
Beaulieu et al [44], 2020	AI^g^ interventions for opioid use	Review of the gray literature to identify AI interventions specific to opioid use disorders being developed, implemented, and evaluated	Mention of HopeBand (prototype for wrist-mounted SpO_2_ sensor) and the A2D2 closed-loop device by Dhowan et al [25]	Description of technologies

^a^SpO_2_: saturation of peripheral oxygen.

^b^SIF: supervised injection facility.

^c^OD: overdose.

^d^PXD: patient experience design.

^e^ECG: electrocardiogram.

^f^mHealth: mobile health.

^g^AI: artificial intelligence.

#### OD Alert Devices

There were 39% (9/23) of papers [26,31-33,35,36,44-46] that either evaluated or described devices designed to monitor vital signs of people who use drugs, such as respiratory rate, SpO_2_, movement, temperature, or heart rate, and prompt an alert once a certain threshold for those vital signs has been reached, indicating that the person might be having an OD. Table 1 depicts the vital signs used by the different devices to detect OD.

#### OD Response Devices

There were 43% (10/23) of papers that either evaluated or described technologies that focused on alerting potential responders to an OD and facilitating a response [26,27,29,30,34,37,40,45-47]. These devices were mostly based on smartphone apps that connect someone witnessing an OD with potential responders. The main purpose is to send an alert of an OD in a close location to someone who can attend, which usually involves being in possession of naloxone or having the capacity to administer it. This would allow responders to act before EMS are able to arrive. In this category, we also included a study by Tukel et al [30] where they test the time to scene taken by drones carrying naloxone.

#### Combined Alert and Response

In total, 48% (11/23) of the papers described devices where both alert and response are linked [24-26,28,38,41-46]. This is also the case for commercial products found in the gray literature [50,52-54]. OD detection devices need to be connected to an interface that allows potential responders to see when and where an OD is taking place. This applies both to sensor devices and supervised consumption devices where, instead of a sensor, the detection device consists of checking on the user through a set of prompts, which are either produced automatically by a smartphone app [41,50,51] or by a human supervisor at the other end of the line [51,55]. There are some devices being developed that attempt to cover both functions by acting as a closed-loop system. In this case, the device uses a sensor that, if detecting an OD, sends a signal to an automatic antidote dispenser attached to the body in the form of either an injection [24,39,45,48,49] or an implant [25]. At present, the studies we identified are in the prototype phase and are more focused on the antidote dispenser than on the sensor. A total of 9% (1/11) of the studies simply suggested such a device as a concept to assess its acceptability in a survey [38].

### Themes of Studies and Contributions

Our thematic analysis resulted in the following themes: acceptability, effectiveness, device use and decision-making, and description of available devices.

#### Acceptability

In total, 30% (7/23) of the studies focused mainly on the acceptability of the technologies described among their (potential) users. Ahamad et al [36] assessed the willingness of people who use opioids to wear a hypothetical skin patch that can detect and alert others of an OD by conducting a survey of 1061 participants, performing logistic regression to assess factors associated with willingness to wear the device. They found a high level of acceptance, with more than half (53%) of their sample of people who use opioids willing to use a wearable device. From their logistic regression analysis, factors associated with willingness to wear the device were having overdosed, currently being on methadone treatment, being a woman, and a history of chronic pain. Homelessness, in contrast, was negatively associated with willingness to wear the device.

Marcu et al [27] investigated the user requirements for a smartphone app to coordinate layperson administration using a qualitative approach through semistructured interviews and focus groups. They concluded that trust-based considerations for the design of smartphone apps to facilitate layperson response will be critical for their adoption and use in real OD situations. Tsang et al [26] carried out a survey among 443 people who use drugs to assess their willingness to use a smartphone monitoring app to mitigate OD. They also asked if respondents would be willing to use an app to report “tainted” (ie, adulterated) drug supply and receive OD alerts from others. They found that ownership of mobile phones and data plans among their sample of people who use drugs was inconsistent. There was a high level of willingness to use apps, especially an app to receive OD and drug supply alerts, among those who had phones and internet. Over half were willing to use smartphone cameras to monitor OD. Nearly three-quarters (73%) were willing to use a bystander alert app for naloxone. Barriers to using the device were discomfort, safety and privacy issues, and being transient. Carlile and Sunshine [33] studied the capacity of people who use opioids to turn off a smartphone simulation of an OD alarm. This alarm would be sent to the user suspected of having an OD to check that they are conscious. They did this through an experimental approach, testing 50 people who use opioids on their ability to turn off such an alarm after opioid use in a supervised consumption facility and a survey of the same group of participants on their acceptance of false alarms. They found that >90% of their sample was able to interact with a smartphone in the minutes following self-injection. The authors concluded that it would be reasonable to expect users to be able to turn off a smartphone alarm if they were not experiencing life-threatening respiratory depression. This shows that using an app to send prompts every set number of minutes to check if the user is responsive could be an effective way to monitor whether users are conscious. Bristowe et al [42] presented a protocol for a pilot study of a supervised consumption line, with uptake measured as the number of calls to the line as the primary outcome. Secondary outcomes included patterns of use of the line and outcomes from the calls, such as EMS dispatches as a result of calls. The pilot was planned for 15 participants who self-disclose as people who use drugs unobserved. The intervention received substantial community support, suggesting that it is an acceptable intervention; however, there are no study results available yet. A study by Kanter et al [38] interviewed 97 people who use opioids to explore their willingness to wear different hypothetical devices, with a special focus on a closed-loop device that would detect an OD and automatically administer naloxone. The questionnaire consisted of Likert scales to assess how likely users were to use different devices according to functionality, and a semistructured interview was used to find out preferences regarding the closed-loop device. The authors found that most respondents to their survey were willing to wear any device proposed that would sense vital signs or an OD or alert bystanders. In total, 67% of respondents were willing to wear a device that sensed an OD and administered naloxone if needed. In terms of the preferred location of the sensors, a watch-appearing bracelet (77%) and a wrist-like bracelet (73%) were preferred. From their semistructured interviews, they identified comfort and discreetness as the most important factors for the device’s appearance, whereas for some, the lifesaving potential of such devices made appearance irrelevant. Reported negative sentiments about such devices included fear of losing a high because of the device injecting naloxone unnecessarily. Other concerns referred to making law enforcement suspicious of such devices, making the person overdosing vulnerable to other ill-intended bystanders, or the device being stolen or lost.

#### Efficacy or Effectiveness

In total, 22% (5/23) of the papers dealt with the effectiveness of different tools or interventions. Nandakumar et al [35] tested the diagnostic accuracy for OD of their Second Chance smartphone sensor, which uses sonar technology to measure breathing rate. This is a proof-of-concept contactless system that converts the phone into a short-range active sonar using frequency shifts to identify respiratory depression, apnea, and gross motor movements. They performed testing in two environments: (1) an approved supervised injection facility (SIF) and (2) an operating room (OR) where they simulated rapid, opioid-induced OD events through general anesthesia. The authors found the device to perform well both in the SIF and the OR. In the SIF, it identified opioid-induced central apnea with 96% sensitivity and 98% specificity and respiratory depression with 87% sensitivity and 89% specificity. In the OR, the device identified 19 of 20 simulated OD events. They concluded that this technology, once coupled with a response app for alerting friends and family carrying naloxone, could have potential as a harm reduction intervention. Singh et al [31] tested the diagnostic accuracy of an algorithm to detect whether users of a wearable sensor (Empatica E4) were cheating in opioid surveillance. In this case, cheating is referred to as collaborative nonadherence (CNA), which translates into giving one’s biosensor to someone else when surveillance is ongoing. The principal aim of this study was to leverage accelerometer and blood volume pulse measurements from a wearable biosensor and use machine learning to detect CNA. To test this, they used quantitative data from 11 patients who had had an opioid OD and cut and pasted snippets from other patients to see whether observers could detect the different physiological signals. The results found a high detection level of CNA, with an average detection accuracy of >90% when the collaborator was one of the patients in the data set of study participants and >86% when the collaborator was from a set of 14 users whose data had never been seen before by the researchers classifying cases. A total of 9% (2/23) of the studies tested hypothetical interventions using historical data. Tukel et al [30] tested the potential of drones to deliver naloxone to an OD scene. They flew a drone to hypothetical OD scenes based on real historical EMS data and compared the drone’s arrival times with the time the EMS took to arrive. They showed that a drone could travel several ranges of straight-line (ie, “as the crow flies”) distance faster than an ambulance took to arrive to the same point. The authors consider that the use of drones to carry naloxone to a person having an OD can be a way to improve the timeliness of responses. Schwartz et al [40] compared emergency response through the use of an emergency response community app against EMS arrival for OD responses based on different parameters. They tested whether equipping community members, including people who use drugs, with a smartphone app enabling them to signal and respond to suspected OD would support naloxone administration in advance of EMS. They also analyzed the number of false and true alarms for administering naloxone.

Khalemsky and Schwartz [37] created a simulation of different location-based alert and emergency responses via a hypothetical responder app, among which was naloxone provision, and compared it with historical data of OD response times by EMS across the United States. They found that an emergency response community would be especially effective in metropolitan areas with a high population density. In this case, average probability of arriving faster than EMS was 30.6%, whereas for the total of responses in different population density areas, it was 23.8%.

#### Device Use and Decision-making

A total of 13% (3/23) of the studies focused on the type of use or heuristics for decision-making among device users. Ataiants et al [29] and Khalemsky and Schwartz [37] looked at the decision-making processes that determine whether an OD response app user would choose to signal an OD or be alerted of OD episodes. Researchers conducted in-depth interviews with 18 participants with varying levels of opioid use and analyzed the data generated by the app used (UnityPhilly) to assess the link between signaling and attending alarms and other variables, namely, gender, opioid use, OD history, and OD witness history. They used thematic analysis to identify decision-making heuristics for the type of signaling and response preferences of users. In total, 3 heuristics appeared: unconditional signaling (“Always signal for help or backup”), conditional signaling (“Rescue, but only signal if necessary”), and conditional responding (“Assess if I can make a difference”). They also found that, among other factors, self-efficacy to administer naloxone and a “helper” identity may contribute to the use of certain heuristics.

Bardwell et al [28] studied the use of a button-based device (Brave Button) intended for OD response in a supported housing facility for women. They interviewed 14 women about their use of the device and found that they used the alert button function more often to alert of violence than of OD episodes. On the basis of this, they highlighted the need to further develop OD detection technologies and the need for technologies to prevent gender-based violence in supported housing. Roth et al [32] looked at the usability of a wearable patch (Spire) attached to clothing to measure respiratory rate and motion and with potential for detection of OD. They recruited 16 individuals who reported ≥4 daily opioid use events within the previous 30 days. They measured hours of recording per day and compared the recordings based on types of users. They found that using a wearable biosensor to monitor physiological changes associated with opioid use was feasible. However, they concluded that more sensitive biosensors that facilitate triangulation of multiple physiological data points and larger studies of longer durations are needed.

#### Description of Devices

In total, 67% (4/6) of the papers under this theme were literature reviews. One paper by Beaulieu et al [44] was a descriptive review of the gray literature on artificial intelligence interventions for opioid use. It included other aspects such as treatment and prevention but also OD detection and response technologies, namely, Second Chance (sonar-based smartphone sensor), HopeBand (wrist-mounted SpO_2_ sensor), and A2D2 developed at Purdue University (closed-loop OD detection through electrocardiogram sensor and naloxone administration through implant). They subdivided the technologies found into 5 categories: smartphone apps (n=12), health care data–related interventions (n=7), biosensor-related interventions (n=5), digital- and virtual-related interventions (n=2), and “other” (ie, those that could not be classified into the other categories; n=3).

The paper by Fairbairn et al [45] is a commentary reviewing the literature to analyze past and current trends in community-based opioid prevention. Among the different interventions described, it mentioned the smartphone OD response apps Beacon Dispatch by Trek Medics and OD Help (now Brave app by Brave Coop). The authors proposed integrating the novel technologies described in their review into community programs to reduce harm in an evolving opioid market. Vilardaga et al [47] conducted a scoping review of gray literature on the internet regarding opioid-related smartphone apps. The paper characterized the purpose, audience, quality, and popularity of 59 opioid-related smartphone apps. There were 2 apps whose purpose was to respond to opioid OD through timely provision of naloxone. These were NaloxoFind and UnityPhilly, both of which are OD response apps linking people with naloxone to those in need. However, NaloxoFind is only for informative purposes, presenting which establishments provide naloxone in different US states. Only UnityPhilly has the capacity to provide the geolocation of an OD incidence and facilitate a response. The authors found that opioid-related apps available to consumers addressed key stakeholders (patients, providers, and community) to be consistent with prevention, treatment, and OD strategies to address the opioid crisis. However, they also noted that there was little evidence that available opioid-related apps met basic quality standards, and no relationship was found between app quality and popularity. They also emphasized that the review was conducted at the level of consumer decision-making (ie, the app store), where quality standards besides user evaluations could only be assessed for very few opioid-related apps. Nonetheless, they highlighted the potential of smartphone apps as a critical tool to increase access to and use of opioid prevention, treatment, and recovery services.

A study reviewed wearable sensors and wireless technology for substance use disorders, including alcohol, cocaine, opioids, and general substance use [46].

A total of 33% (2/6) of the devices were listed as opioid OD sensors: Second Chance, the sonar-based smartphone sensor described by Nandakumar et al [35], and the Empatica E4 wrist-mounted sensor, citing a study by Chintha et al [57] in which it was used to measure opioid toxicity after naloxone administration. In this case, the E4 was not used in the study as a tool to sense an OD but to measure physiological changes after an OD has been attended, and therefore, this study does not meet our inclusion criteria. Nonetheless, it is noteworthy that this device has been used to measure these physiological changes as it gives an indication of what physiological indicators the device will be able to measure in case of OD. The E4 wristband was also used in the aforementioned study on CNA [31]. Wearable sensors were the most commonly used technology, with OD monitoring devices being among them. The study highlights the advantages of new technologies over traditional therapies by increasing geographic availability and continuously providing feedback and monitoring while remaining relatively noninvasive.

In total, 33% (2/6) of the studies were limited to describing proof-of-concept devices. Imtiaz et al [43] described their design for a closed-loop device. Once the sensor detects that the user’s SpO_2_ level is <90%, it triggers a response that consists of a subcutaneous administration of the opioid antidote nalmefene and transmission of a GPS-trackable 911 alert. Bivens [41] described OD Help as proof-of-concept and analyzed its capacity to prevent OD in the framework of “patient experience design” as a model for integrating user experience into technology development. This study proposed the integration of the OD Help app with a commercial breathing sensor (Spire).

In addition to device descriptions in academic literature, we found commercial products with company descriptions of them on their websites. Carezapp [53] developed a system intended for use in shelters and assisted accommodation for people who use drugs that they are piloting with charities in the United Kingdom. It uses radar sensors to monitor vital signs. It consists of 2 sensors, one to go on the ceiling and the other to go under the bed that measure respiratory rate and heart rate. It also includes a tablet device that is locked to a preinstalled web application and a Wi-Fi router. For example, sensors monitor an individual’s breathing rate when they are in their accommodation. If it falls below what is normal for them, the system will notify the staff, who can then go and check on the person. Brave Coop has developed the Brave app, which allows people who use drugs to connect with trained volunteers who follow a protocol to make sure that the person is well while they are using drugs and alert agreed responders in case of OD. This app is being used in Canada, the United States, and recently the United Kingdom. The Brave Sensor, also developed by Brave Coop, is a system that alerts responders in the event of an OD in an enclosed space such as a washroom [52]. The Brave Sensor uses radar, only monitoring motion. If it detects stillness, the system instantaneously alerts by sending an SMS text message to the designated responder’s phone. SafetyNet by Masimo was designed for the home care of patients taking opioid medications. It consists of a bracelet wired to a finger sensor for pulse oximetry and heart rate and is connected via Bluetooth to a home hub, which then connects to the individual’s smartphone and to designated responders. Its integrated app provides escalating alerts (ie, “caution,” “warning,” and “emergency”) when SpO_2_ levels drop [56].

Finally, the 3 projects identified that have been awarded National Institutes of Health grants are developing closed-loop systems for OD detection and naloxone administration. The key components of the system presented by BioSensics LLC are (1) a wrist-worn device that detects respiratory arrest based on measures of SpO_2_ levels, respiration rate, and heart rate; (2) a medication delivery device affixed to the thigh using biocompatible adhesive patches that automatically delivers naloxone intramuscularly when respiratory arrest is detected; and (3) a companion iOS or Android app that will be installed on the user’s smartphone to contact an emergency call center with the user’s GPS location when the system is triggered [48]. Both other projects by Narayan [39] and Mackie and Guo [49] present a closed-loop system in which a wearable sensor can track variations in physiological parameters (SpO_2_, heart rate, and breathing pattern) and deliver naloxone subcutaneously through microneedle patches, a delivery method only shared by these 2 projects.

## Discussion

### Principal Findings

In this review, conducted systematically, it is clear that there is a broad variation in technology-led devices that are being used to identify and intervene in a timely and effective manner when individuals experience a potentially fatal OD. There has been increasing interest, especially after the COVID-19 pandemic, in the application of these technologies.

This recent emergence of research in this area marks a consolidation of the technologies needed to provide adequate data coupled with a continued increase in drug ODs, especially in North America, where highly potent opioids have become the norm, but also in Europe, particularly in the United Kingdom and other countries in Northern Europe where DRDs have increased in recent years [5]. This situation has prompted governments to examine and fund new approaches to tackle drug ODs. An example is the Scottish Government’s Health and Industry Partnership, which has established a tackling drug deaths consortium. Its purpose is to bring together experts in substance misuse with industry partners, supporting the national mission to reduce drug harms and promote recovery, tackling the drug death emergency as a key priority area for innovation in Scotland.

Reflecting this situation, all initiatives that we found were based in North America or referred to the North American context, although we found in the gray literature companies developing ODART operating in Scotland [51,53,54] and 1 company operating ODART in several other countries for prescribed opioids in home settings [56].

The highest rates of drug deaths in most countries with high OD rates occur among middle-aged men [2,3,5,10]. Any solution should consider that many people who use drugs in the higher age ranges are not highly digitally literate [58]. This should not deter researchers and harm reduction services from providing technical solutions but highlights the fact that they should be tailored to individuals and provided along with appropriate training. ODART interventions, whether they are related to supervised consumption, responder apps, or wearables, will most likely require users to have access to smartphones and data. However, many people who use drugs will not carry a smartphone on a regular basis and may have limited data plans, if at all [26]. Any piloting or implementation of these interventions should ensure that these needs are met. The capacity of a device to be comfortable, discrete, and trustworthy and guarantee safety and privacy will also be of great importance [26,27,38].

In Scotland, most deaths occur at the person’s home or at someone else’s home [10]. Statistics also show that a large proportion (more than half in some countries) of drug deaths happen when the person is alone [3,6], which highlights the importance of remote ODART interventions. To prevent OD from occurring indoors, solutions such as supervised consumption devices and wearables might be the most effective. Nonetheless, many people use drugs and OD outdoors. In this case, technologies to improve timely response involving bystanders and the broader community are likely to have the most impact.

Many of the devices described show promising features that could allow for broader implementation. Simulation studies suggest that community responder networks and drones are likely to arrive earlier to an OD site than EMS [30,37]. The fact that naloxone might be all that a potential responder needs to reverse an OD makes the use of drones feasible for these lifesaving interventions. However, more real-life effectiveness research is needed to test whether assumptions for simulation models hold in real-life scenarios. An important finding in this regard is that ODART—in this case, responder apps—will be most effective in metropolitan areas where the density of people carrying naloxone and who have access to an app is sufficient [29,37]. This essential condition is highly dependent on public policies and awareness campaigns that must accompany the implementation of technologies. Further research is warranted to find out more about how naloxone can be deployed through drones and community members and the ethical and legal implications.

Knowing the characteristics of those who accept (or not) to use ODART within each population will be an important factor for implementation, allowing for the tailoring of interventions and direct efforts to include those who could benefit from them but might be reluctant, such as homeless people who use drugs, as was found by Ahamad et al [36]. This can be a challenge for implementation as this is one of the most vulnerable groups of people who use drugs and they are at high risk of OD [38]. In contrast, the same study found that those with a previous history of OD were willing to wear ODART devices, a valuable insight for future interventions as having a history of OD is a risk factor for further OD [36].

Overall, the research described in our review seems to support the idea that people at risk of OD and community members are willing to make use of ODART, although most studies on acceptability (4/7, 57%) relied on interviews about prospects of use [26,27,36,38]. However, some studies (3/23, 13%) analyzed behavioral data, such as the study by Ataiants et al [29], who found that unconditional signaling (ie, making oneself available for rescue backup in any situation) was carried out frequently by people who use opioids, an encouraging finding that stands in contrast to previous research indicating that reluctance to call 911 has been a major deterrent to prevent OD deaths in the United States [59].

We classified ODART into “alert,” “response,” and “combined alert and response” technologies. Currently, most devices described in the academic literature are more focused on either the alert or response functions. Combined functionality will increase once these technologies are further developed and comprehensive systems appear. We can place the technologies described in a range of low technology to high technology, where devices such as apps or phone lines to supervise consumption would be at the low-technology end, other technologies such as sensors would be in the middle range, and closed-loop devices would be at the high-technology end. The level at which different devices sit across this spectrum is likely to determine the cost and the time it takes for these interventions to be implemented, with high-technology devices more likely to be more costly, at least in the first stages, and take longer to develop and be commercialized. At the same time, devices developed by industry that are already being used generally lack evaluations published in academic literature. Furthermore, different individuals will have different preferences, and no single device will be acceptable to all people who use drugs, so offering them a variety of options will be critical to the uptake and subsequent effectiveness of these interventions. Therefore, it is advisable to pilot different options, publish results, and start implementing low-technology devices without delay if they are readily available and have the potential to save lives. In the long run, high-technology devices can be integrated into existing systems that lower-technology devices have laid the groundwork for.

Notably, only 4% (1/23) of the studies mentioned nalmefene instead of naloxone as an antidote for opioid OD, arguing that the necessary dose is lower and it could be more effective with potent synthetic opioids [43]. This also warrants further research and discussion among the medical community.

### Limitations and Strengths of This Review

This study is unique in its mapping of current trends in the development of ODART. It covers a vast area and a long period. We chose to conduct our search in MEDLINE and not the entire PubMed database, which might be a limitation regarding the number of articles found. However, our secondary search does not suggest that we missed any important papers. As the development of ODART is very recent, published research is scarce, and most papers in this review described devices in a proof-of-concept or prototype state. One of the issues for identifying ODART is that different technologies that have been developed by the commercial sector have not been published in peer-reviewed papers. In contrast, the different technologies that have been introduced by research studies have never been implemented in real settings. In addition, research and development at this stage are dominated by North America, which might not reflect other contexts and drug use patterns. Reflecting this, all studies referred to OD caused by opioids, which is the main concern in these countries. Other countries might also be concerned by other substances causing or contributing to OD, such as benzodiazepines, GABAergic substances, or stimulants such as methamphetamine, or DRDs caused by cocaine-induced cardiac effects [4]. Moreover, some studies presented (9/23, 39%) were conducted by just a few groups, with 6 of them headed by a group led by researchers largely based in Philadelphia, Pennsylvania, United States, and Drexel University accounting for 26% (6/23) of the papers [27,29,32,34,38,40] and a group led by researchers at the University of Washington accounting for 13% (3/23) of the papers [24,33,35]. More research on user needs and preferences from other countries and on other substances is encouraged, and new literature reviews should be conducted in a few years to update the state of the art. Our review might not reflect the whole spectrum of devices that are able to detect and alert of a drug OD as there is a myriad of clinical devices already operating, either in hospitals or in the context of “hospital at home,” that monitor vital signs and either can or have the potential to alert responders. We intentionally excluded technologies that were not specific to drug OD to keep a narrow scope and focus on technologies that are more likely to be used in the context of illicit drug use and misuse of prescribed drugs in the future. Similarly, we excluded devices that had only been documented in patents. Although it might be useful to describe these devices, we wanted to collect information on technologies that have evidence of being used or developed with the aim of providing a picture of the current state of the art.

### Conclusions

ODART poses a potential solution to rising OD rates in many countries, especially when it comes to the faster administration of naloxone for opioid OD. In the last few years, there has been an increasing interest among the scientific community in the development of these technologies. The available literature indicates that there is also a willingness to use these devices by people who use drugs and affected communities. There is still no definitive technology, either sensor-based or app-based, that is ready for broad implementation. More real-life effectiveness studies are needed to further develop these technologies and adapt them to the different populations that might benefit from them.

## Appendix

Multimedia Appendix 1 Search strategy overdose detection and alert technologies.

Multimedia Appendix 2 Detailed table of studies.

## Abbreviations

CNA: collaborative nonadherence
DRD: drug-related death
EMS: emergency medical services
OD: overdose
ODART: overdose detection and alert technologies
OR: operating room
PRISMA-P: Preferred Reporting Items for Systematic Reviews and Meta-Analyses extension for Protocols
SIF: supervised injection facility
SpO_2_: saturation of peripheral oxygen
