# Supplementary material for: Overdose Alert and Response Technologies: State-of-the-art Review
Source: J Med Internet Res. 2023 Feb 15;25:e40389. doi: 10.2196/40389 (PMC9978985; doi:10.2196/40389)
Supplement: Multimedia Appendix 1 [file jmir_v25i1e40389_app1.docx]

Search strategy

Databases:

Medline

Embase

Web of Science

Scopus

ACM

IEEE

SciELO

Dates 1st January 2010

All searches run 6/4/21 and 6/03/22

Example search from Ovid MEDLINE(R) ALL <1946 to June 03, 2021>

1 Remote Sensing Technology/

2 Telemedicine/

3 Wireless Technology/

4 Monitoring, Physiologic/

5 Real-time remote monitoring.tw.

6 Telehealth.tw

7 Telemedicine.tw.

8 Telemetry/

9 Smartphone/

10 exp technology/

11 mobile health.tw.

12 Telehealth.tw.

13 telemedicine.tw.

14 (ehealth or e-health).tw.

15 (mhealth or m-health).tw.

16 ("telebehavio?ral health" or "tele care" or telecare or "tele coaching" or telecoaching or telecomm* or tele-comm* or "tele conference*" or teleconference* or "tele consultation" or teleconsultation or "tele health care" or "tele health*" or telehealth* or tele-health or "tele management" or telemanagement or "tele med*" or tele-med* or "tele mental health*" or "telemental health*" or telemetry or tele-monitor* or telemonitor* or telepractice or "tele practice" or tele-psych* or telepsych* or "tele speech" or telespeech or "tele therap*" or tele- therap* or teletherap*).tw.

17 mobile applications/ or software design/ or exp user-computer interface/

18 ((app or apps or application*) adj2 (smartphone* or smart-phone* or mobile* or phone* or sensor* or software)).tw.

19 wearable electronic devices/ or fitness trackers/

20 (smartwatch* or (wearable adj device*) or wearables or "real-time monitoring device*" or actigraphy or accelerometer*).tw.

21 ("digital media" or "software program*").tw.

22 ((Internet or digital* or online* or on-line or web* or virtual) adj2 (deliver* or information or communication* or assisted or e-learning or support)).tw.

23 exp Drug Overdose/ or overdose.tw.

24 poison*.tw.

25 or/1-22 655249

26 23 or 24

27 25 and 26

28 limit 27 to yr="2010 -Current"
