# Supplementary material for: Overdose Alert and Response Technologies: State-of-the-art Review
Source: J Med Internet Res. 2023 Feb 15;25:e40389. doi: 10.2196/40389 (PMC9978985; doi:10.2196/40389)
Supplement: Multimedia Appendix 2 [file jmir_v25i1e40389_app2.docx]

|  | **Authors** | **Title** | **Intervention** | **Function** | **Focus** | **Device** | **Theme** | **Study design** | **Journal** |
| --- | --- | --- | --- | --- | --- | --- | --- | --- | --- |
| 1 | Ahamad et al., 2019 [36], 2019 | Factors associated with willingness to wear an electronic overdose detection device | Wearable biosensors | OD alert | Willingness of PWUD to wear a device (skin patch) that can detect and alert others of an overdose | Hypothetical app using smartphone cameras to monitor breathing while using drugs | Acceptability | Quantitative (survey), PWUD (n = 1061) were willing to wear a device to detect overdose in Canada | Addiction Science and Clinical Practice |
| 2 | Ataiants et al [29], 2021 | Decision-making by laypersons equipped with an emergency response smartphone app for opioid overdose | Responder app | OD response | Identification of heuristics that determine whether someone with a response app will signal an OD or be alerted of OD episodes or not based on need for assistance and contextual information. | Smartphone OD response App: UnityPhilly | Usage/Decision-making | Qualitative, In-depth interviews, app data analysis with PWUD (n= 8) and non-PWUD (n= 10) at the US | International Journal of Drug Policy |
| 3 | Bardwell et al [28], 2021 | Women's multiple uses of an overdose prevention technology to mitigate risks and harms within a supportive housing environment: a qualitative study | Alarm button to alert staff in building | Combined alert and response | Uses for OD alert device by women in supported housing for women | OD response button (Brave button) | Usage/decision-making | Qualitative (Semi-structured interviews) with PWUD (n=14 cisgender women) in Canada | BMC Women's Health |
| 4 | Beaulieu et al [44], 2020 | Artificial intelligence interventions focused on opioid use disorders: A review of the grey literature | AI interventions for opioid use | Different technologies 1. OD alert 2. Combined alert and response | Review of the grey literature to identify AI interventions specific to opioid use disorders being developed, implemented and evaluated | Mention to 1. Second Chance (sonar-based smartphone sensor); Hopeband (prototype for wrist-mounted SPO2 sensor); Empatica (wrist-mounted heartbeat, motion, skin electrical conduct, and temperature sensor) but as withdrawal detector, not OD; 2. A2D2 Purdue (closed loop OD detection through EKG sensor and naloxone administration through implant) | Description of technologies | Descriptive review (grey literature review, n=29) focused on PWUD, undertaken in Canada but with international scope | The American Journal of Drug and Alcohol Abuse |
| 5 | Bivens [41], 2018 | Reducing harm by designing discourse and digital tools for opioid users' contexts | Smartphone app to use naloxone | Combined alert and response | Extend Patient Experience Design (PXD) to include community-based and technology-based contexts of use by analysing two case examples and discuss implications of PXD. Suggesting contexts of use-heuristic categories to consider when designing health communication information for users in these contexts | Mention to OD response smartphone app (OD Help) and potential combination with OD detection skin patch sensor (Spire) | Description of technologies. Implications of community-based and technology-based | Qualitative, PWUD in US | Communication Design Quarterly |
| 6 | Bristowe et al [42], 2021 | Virtual overdose response for people who use opioids alone: protocol for a feasibility and clinical trial study | Supervised consumption service | Combined alert and response | Protocol for piloting a telephone-based supervised opioid consumption service | Supervised Consumption line | Acceptability | Protocol for clinical trial study to pilot a telephone-based supervised opioid consumption service – PWUD alone (n=15) in Canada | Journal of Medical Internet Research – Research Protocols |
| 7 | Carlile and Sunshine [33], 2019 | Ability of opioid-impaired users to interact with a smartphone | Smartphone sensor and alert | OD alert | Testing opioid user’s ability to turn off smartphone simulation of an OD alarm and survey on acceptance of false alarms | Prototype smartphone OD alert app that would send a prompt to check on user alert | Acceptability. Ability to turn off false alarms | Quantitative (Measurement of response to alarm in the single group plus survey with the same group) – PWUD (n =50) in Canada | Journal of Investigative Medicine |
| 8 | Chan et al [24], 2021 | Closed-loop wearable naloxone injector system | Closed loop device formed by sensor and naloxone injector | Combined alert and response | Present proof of concept and evaluated in two environments: Supervised injection facility (SIF) and hospital environment simulating opioid induced apnea in healthy participants | closed-loop wearable injector system that measures respiration and apneic motion using a pair of on-body accelerometers, and administers naloxone subcutaneously upon detection of an apnea | Evaluation of accuracy and efficacy of device | Quantitative. clinical trial (no control group and not blinded) PWUD supervised injection facility (n=25) in US and Canada | Scientific Report - Nature |
| 9 | Dhowan et al [25], 2019 | Simple minimally-invasive automatic antidote delivery device (A2D2) towards closed-loop reversal of opioid overdose | Closed-loop device formed by sensor and naloxone injector | Combined alert and response | Present proof of concept and tested leakage in lab simulation (beakage with saline solution) and release capacity in mice | Subcutaneously placed device can be activated using an externally applied time-varying magnetic field from a wearable device. The device would be paired to a ECG and respiratory rate sensor | Proof of concept. Testing of release capacity in mice | Quantitative. In vivo and in vitro evaluation of device capabilities (3 animals - mice) in the US | Journal of Controlled Release |
| 10 | Fairbairn et al [45], 2017 | Naloxone for heroin, prescription opioid, and illicitly made fentanyl overdoses: Challenges and innovations responding to a dynamic epidemic | Different interventions, including apps and sensors | Different technologies. OD response and combined alert and response | Overview of trends in opioid use in North America and proposing potential solutions | Smartphone OD response apps: Beacon Dispatch by Trek Medics and OD Help (now Brave App) | Description of technologies | Review - Essay, literature review on PWUD in the US | International Journal of Drug Policy |
| 11 | Goldfine et al [46], 2020 | Wearable and Wireless mHealth Technologies for Substance Use Disorder | Digital interventions for substance use. Wearable sensors and wireless technology | Different technologies. Alert, alert | to evaluate the advances in wearable and other wireless mobile health (mHealth) technologies in the treatment of substance use disorders. | Nandakumar et al sonar-based smartphone sensor (Second Chance) Wrist mounted sensor (Empatica E4), Ahmad et al paper on hypothetical sensor, Dhowan et al A2D2 closed-loop device | Description of technologies | Scoping review (n=28) on wearable sensors and wireless technology for treating PWUD, undertaken in the US but with international scope | Current Addiction Reports |
| 12 | Imtiaz et al [43], 2021 | Hypoxia driven opioid targeted automated device for overdose rescue | Closed-loop device formed by a sensor and nalmephene injector | Combined alert and response | Presents design of closed-loop device with sensor for SPO2 and subsequent release of naelmephene when SPO2 goes under 90% | Wearable non-invasive closed-loop device | Description of technologies | Proof of concept in the US | Scientific Reports – Nature |
| 13 | Kanter et al [38], 2021 | Willingness to use a wearable device capable of detecting and reversing overdose among people who use opioids in Philadelphia | Wearable sensors that detect and prompt an automated response. Different functionalities offered but with special interest in closed-loop device that deploys naloxone | Combined alert and response | Willingness of PWUDs to wear a device (wrist band) that can detect an OD and prompt response. Different options are given and open questions allow for respondents input | Hypothetical wearable to detect an OD and elicit response with different options | Acceptability | Mix method (qualitative and quantitative) Survey and semi-structured interviews with PWUD (n=97) in the US | Harm Reduction Journal |
| 14 | Khalemsky and Schwartz [37], 2017 | Emergency Response Community Effectiveness: A simulation modeller for comparing Emergency Medical Services with smartphone-based Samaritan response | Responder app. Community response based on geolocation app | OD response | Simulation of different emergency responses, among which naloxone provision for OD. Comparing a simulation of time to respond based on real-world data of community response vs EMS based on different parameters and their probability | Hypothetical smartphone OD response | Effectiveness. Simulation of Emergency Response Community effectiveness compared to Emergency Medical Services | Quantitative. PWUD and community members in the US (analysis in Israel) | Decision Support Systems |
| 15 | Marcu et al [34], 2020 | Empowering communities with a smartphone-based response network for opioid overdoses | Smartphone apps to connect responders. Witness of OD | OD response | Piloting a smart app to connect responders to each other. UnityPhilly is intended to create a network of PWUD, as well as other members of the local community who report they have not had any non-medical opioid use. | Smartphone OD response App: UnityPhilly | Feasibility | Quantitative (Pilot-survey) PWUD and other community members of who report they have not had any non-medical opioid use in the past 30 days (n= 57, n=55) in the US | IEEE Pervasive Computing |
| 16 | Marcu et al, 2019 [27] | Acceptability of smartphone applications for facilitating layperson naloxone administration during opioid overdoses | Smartphone apps to connect responders | OD response | User requirements for a smartphone application to coordinate layperson administration of naloxone during an opioid OD | Hypothetical smartphone OD response | Acceptability | Qualitative (Interviews and focus groups) – PWUD and other community members (n=19) in US | Journal of American Medical Informatics Association (JAMIA) |
| 17 | Nandakumar, N. et al., 2019 [35], 2019 | Opioid overdose detection using smartphones | Smartphone sonar sensor | OD alert | Test algorithms that run on smartphones and unobtrusively detect opioid OD | Sonar based smartphone sensor (Second Chance) | Efficacy of tool (experiment) | Quantitative (RCT) PWUD (n =94) in US | Science Translational Medicine |
| 18 | Roth, A. et. al., 2021 [32], 2021 | Wearable biosensors have the potential to monitor physiological changes associated with opioid overdose among people who use drugs: A proof-of-concept study in a real-world setting | Wearable sensor | OD alert | Usability of wearable patch attached to clothing measuring respiratory rate and motion. Hours of recording per day and comparison of recording based on types of user. Potential for detection of OD | OD Help (then Be Safe now Brave app) | Feasibility. Potential for wearable sensor to be worn by PWUO and to record appropriately | Quantitative (Each participant was assigned a wearable biosensor that measured respiratory rate (RR) and actigraphy every 15s for 5 days and also completed a daily interview assessing drug use.), PWUD (n = 16) in the US | Drug and Alcohol Dependence |
| 19 | Schwartz, D. G. et al., 2020 [40], 2020 | Layperson reversal of opioid overdose supported by smartphone alert: A prospective observational cohort study | Smartphone apps to connect witnesses of OD with responders | OD response | To investigate whether equipping community members, including PWUD, with a smartphone application enabling them to signal and respond to suspected overdose would support naloxone administration in advance of Emergency Medical Services (EMS) | Smartphone OD response App: UnityPhilly | Effectiveness of tool (real life) | Quantitative (Pilot) from PWUD (n=57) and community members (n=55) in the US | eClinical Medicine |
| 20 | Singh et al [31], 2019 | A Machine Learning-based Approach for Collaborative Non-Adherence Detection during Opioid Abuse Surveillance using a Wearable Biosensor | Wearable sensor | OD alert | To leverage accelerometer and blood volume pulse measurements from a wearable biosensor and use machine learning for the novel problem of collaborative non-adherence detection in opioid surveillance | Wrist mounted sensor (Empatica E4) | Effectiveness of tool. Diagnostic accuracy in detecting cheating | Quantitative (Data extraction, cutting and pasting snippets from other patients and detection evaluation), (patients’ opioid with overdose, n=11), US | Biomedical Engineering Systems and Technologies |
| 21 | Tsang et al [26], 2020 | Acceptability of technological solutions for overdose monitoring: Perspectives of PWUD | Different smartphone-based solutions to prevent OD | Different technologies OD allert, alert | Willingness to use mobile for monitoring applications to mitigate OD. Also asked about app to report tainted drug supply and to receive OD alerts from others | Hypothetical app to alert bystanders with naloxone | Acceptability | Mix method (qualitative and quantitative) Survey and structured interviews with PWUD alone (n=443) in Canada | Substance Abuse |
| 22 | Tukel, C. A. et al., 2020 [30], 2020 | Time-to-scene for opioid overdoses: are unmanned aerial drones faster than traditional first responders in an urban environment? | Drones for naloxone delivery | OD response | Comparing time required for a drone carrying naloxone to traverse various distances, against the time required for ambulances to traverse similar distances while responding to the scene of actual or suspected opioid overdoses | Modified Dà- Jiāng Innovations (DJI) ‘Inspire 2’ drone to carry naloxone | Effectiveness of tool | Quantitative - use of drone to dispatch naloxone to similation of people with opioid overdose (n=50) and comparison with historical data for ambulance OD emergencies in US | British Medical Journal Innovations |
| 23 | Vilardaga, R. et al., 2020 [47], 2020 | Review of Popularity and Quality Standards of Opioid-Related Smartphone Apps | Opioid-related smartphone apps (no intervention) | Different technologies OD response | characterize the purpose, audience, quality, and popularity of opioid-related smartphone apps. | Smartphone OD response App: NaloxoFind, UnityPhilly | Description of technologies. Quality evaluation | Scoping review and Quantitative (web scraping – opioid-related apps n=61) undertaken in US but international scope | Current Addiction Reports |
